# Sounds easy, looks nice: Crossmodal transfer of auditory processing fluency to visual object preference

**DOI:** 10.3758/s13414-025-03177-5

**Published:** 2025-12-01

**Authors:** Sarah Knight, Jonathan C. Flavell, Sven Mattys

**Affiliations:** 1 School of Psychology, Newcastle University, Newcastle upon Tyne, NE1 7RU UK; 2 Department of Psychology, University of York, York, YO10 5DD UK

**Keywords:** Perceptual fluency, Preference, Crossmodal, Audiovisual

## Abstract

Fluent (i.e., rapid and efficient) processing of perceptual objects is vital for humans to successfully navigate their environments. The experience of fluent processing per se tends to trigger positive affect; however, this affect is often misattributed by perceivers to the objects themselves, meaning that easy-to-perceive objects are judged more positively. These fluency-based preference effects have been robustly demonstrated unimodally, and specifically in situations in which fluency and preference are manipulated and measured for the same objects. However, little is known about how this effect operates crossmodally, when manipulations of fluency in one domain are combined with preference judgements for unmanipulated objects in another domain. In six experiments, we manipulated perceptual fluency in a simple auditory task which participants performed whilst viewing visual objects, which they rated for liking. We found that visual objects presented with easier (more fluent) auditory stimuli received higher liking ratings. This effect persisted when a temporal lag was introduced between the auditory/visual components, but was less reliable for static (as opposed to moving) objects. These results show that fluency-based preference effects can operate crossmodally. They have implications for a range of real-world contexts involving preference and attitude change.

## Introduction

The processing of any given stimulus can be characterised according to a set of general response parameters, such as speed and accuracy. Taken together, these parameters tend to equate to a subjective experience of processing ease, or “fluency” (Reber et al., 2004). Survival in a complex environment requires the rapid detection of relevant target stimuli. Presumably as a result of this evolutionary pressure, rapid and successful target detection is often rewarded by a pleasure response – in other words, fluent processing of a stimulus can trigger a mild positive affect (Winkielman et al., 2003). This pleasure response may then be attributed to the target object itself (Reber et al., 2004), resulting in a general preference for stimuli which are more, as opposed to less, easily processed. We refer to this phenomenon as “fluency-based preference”.

As a result of these fluency-based preference effects, manipulations that facilitate stimulus processing tend to increase liking for those stimuli. Processing fluency can be altered at a conceptual or a perceptual level. Conceptual fluency involves the processing of higher-level information such as semantic knowledge, and can be manipulated by paradigms and features such as semantic priming, rhyme and semantic congruency (Lee & Labroo, 2004). For example, (Whittlesea 1993; Experiment 5) presented participants with target words in either semantically predictive or neutral written sentence contexts, and asked them to judge the words for pleasantness; they found that words presented in the predictable context were judged to be more pleasant than words presented in the neutral context. Of interest for the current study, however, is perceptual fluency. This relates to the effects of lower-level, surface features of target objects on processing fluency. For example, preference for an object can be affected by increased perceptual fluency arising from the number of presentations of that object (the “mere exposure effect”; Mandler et al., 1987; Zajonc, 1968), its duration of presentation (Reber et al., 1998), and its smoothness of motion (Flavell et al., 2019). Similar preference effects are seen for manipulations which facilitate the detection of camouflaged or hidden objects (Erle et al., 2017; Flavell et al., 2020; Muth et al., 2016).

Perceptual fluency-based preference effects have been explored in both the visual and the auditory domains. Typically, processing fluency is assessed through response times or accuracy on a perceptual task involving the stimulus objects, while preference is assessed via liking ratings for those objects (although other metrics of preference, such as self-reported judgements of learning, have also been used, e.g., Rhodes & Castel, 2008). For example, Flavell et al. (2020) manipulated the perceptual fluency of visual objects by changing both the visual contrast of a moving object relative to its background and the degree of camouflage hiding the object. At some point during its trajectory, the object briefly changed size, and participants were required to press a button when they detected the size change. Fluency was assessed via reaction times (RTs), with faster responses to the size change for the higher contrast/less camouflaged objects indicating a successful manipulation of perceptual fluency. Preference was assessed via liking ratings. Higher ratings were obtained for the higher contrast/less camouflaged objects, reflecting perceptual fluency-based preference effects. Similarly, Reber et al. (1998) manipulated the visual contrast of simple shapes (Experiment 2) and the duration of their presentation (Experiment 3), and reported higher prettiness/lower ugliness (Experiment 2) and higher liking/lower disliking (Experiment 3) ratings for the shapes with higher contrast and longer presentation.

Comparable effects have been shown in the auditory domain. For example, Szpunar et al. (2004) reviewed a number of studies showing that an increased number of presentations led to an increase in liking for musical stimuli; they also demonstrated that clips of orchestral music presented more often during a passive-listening exposure phase received higher liking ratings (but not recognition ratings) than clips presented less often. Such effects can even be observed for human voices, where preferences are generally highly complex and idiosyncratic (Lavan & Sutherland, 2023). Babel and McGuire (2015) showed that voices which are more easily categorised according to the self-reported gender of the speaker (i.e., allowed for more fluent processing of gender information) are also rated as more attractive – although the effect of categorisation fluency interacts with stereotypicality judgements to predict attractiveness ratings.

Although perceptual fluency-based preference effects have been demonstrated in the visual and auditory domains separately, it is unclear whether or not they can operate crossmodally. In other words, can the perceptual fluency of a stimulus presented in one modality (e.g., visual) affect preference for a stimulus co-presented in a different modality (e.g., auditory)? On the one hand, it is possible that changes in preference occur only for the stimulus for which perceptual fluency has been manipulated, precluding any transfer to simultaneous, but unmanipulated, stimuli in different perceptual domains. On the other hand, it is possible that the positive affect generated by fluent processing of a stimulus may transfer to other stimuli present in the environment at the same time. This type of crossmodal transfer could be conceptualised as being akin to a “halo effect” (e.g., Nisbett & Wilson, 1977), in which a positive feeling about some aspect of the sensory environment leads to positive judgements about other, unrelated, aspects. It could also be characterised as a form of crossmodal integration, in which information is combined across sensory modalities during perceptual judgements (Bizley et al., 2016).

Some existing work hints at the possibility of crossmodal transfer. Unkelbach (2006, 2007) demonstrated that the manipulation of one variable (e.g., colour contrast) can give rise to fluency-based effects arising from a different variable (e.g., spatial rotation). In other words, learned associations with fluency derived from one stimulus feature can transfer to fluency differences generated by a different stimulus feature. Although Unkelbach’s work was conducted unimodally, it nevertheless suggests that fluency-based effects might transfer across the various aspects of a perceptual scene. Ziembowicz et al. (2013) presented “possible” and “impossible” melodies (according to a previously learned artificial grammar) alongside geometrically possible or impossible visual shapes. They found that the coherence of the pairing (i.e., whether or not both items of a pair were [im]possible) affected participants’ likelihood of reporting either the auditory or the visual stimulus as possible and/or familiar, even when they denied using information from the other modality in making their decisions. It can be assumed that the “possible” stimuli were processed more fluently that the “impossible” stimuli, and it is clear that participants used information from both the auditory and the visual domains when making their decisions. However, the study did not directly test either processing fluency or preference. For instance, participants were not asked to make a timed response to a perceptual feature of the stimuli, nor to provide liking ratings. Shen and Sengupta (2014), meanwhile, found that participants showed greater preference for visual stimuli presented on the same side as an unrelated, to-be-attended auditory stimulus. However, these results point primarily to an enhancement of processing fluency (and hence preference) for the visual stimuli via a spatial auditory cue. This is in contrast to a “true” crossmodal effect, in which enhanced fluency in one domain directly influences preference in another. Suzuki and Gyoba (2008), for example, showed that prior visual exposure to items led to higher preference ratings when those items were subsequently judged by touch (but not vice versa), which they characterise as a crossmodal mere exposure effect. This suggests that fluency-based preference effects may also arise in the audiovisual crossmodal context; however, such effects have yet to be directly tested.

In the current study, we addressed this gap by testing the effect of auditory fluency on participants’ preference for visual objects. Specifically, we extended the task used in Flavell et al. (2020), incorporating an auditory perception task alongside preference judgements for visual objects. Processing fluency was held constant in the visual domain but manipulated in the auditory domain through altering the signal-to-noise ratio (SNR) of a tone-in-noise detection task. Each trial of the auditory task was accompanied by the presentation of a visual object. After each trial, participants were asked to rate their liking for the object. We hypothesised that liking ratings would be higher for objects that were presented concurrently with an easier (i.e., more fluent) auditory task – that is, a higher SNR in the auditory stimuli would result in higher liking ratings for the visually presented objects.

## Experiments

Data were analysed in R (version 4.1.1; R Core Team, 2024), using RStudio (version 1.4.1717; RStudio Team, 2024), and the packages *dplyr* (version 1.1.3; Wickham et al., 2023), *tidyr* (version 1.3.0; Wickham et al., 2024), *lme4* (version 1.1.34; Bates et al., 2015) and *ggplot2* (version 3.4.4; Wickham, 2016).

## Experiment 1: Pilot study

Experiment 1 was a pilot study designed to check the validity of our auditory perceptual fluency manipulation. Participants heard a tone masked by white noise in a variety of SNRs and were required to press a button when they detected a temporary change in the frequency (pitch) of the tone. The SNRs ranged from relatively high (easy) to relatively low (hard). We predicted that perceptual fluency would be greater in the higher SNRs, resulting in the shortest RTs for the highest SNRs and the longest RTs for the lowest SNRs.

### Methods

#### Participants

Due to the well-established basic perceptual link between SNR and target detectability, it was not considered necessary to recruit a large sample of participants to test for an effect of SNR. Instead, a small opportunity sample of participants was recruited to check that the hypothesised hierarchy of RTs was observed (i.e., that the signal processing aspect of stimulus generation had been performed correctly). Fourteen volunteer participants (nine female, five male; mean age = 21 years (SD = 0.784)) were recruited as part of a Master’s project. One participant reported having hearing loss or hearing difficulties; however, that person responded correctly to the audio check trials (see below); also, their responses in the main task were not outliers and followed the trend of the wider sample. Their data were therefore not excluded from the analysis.

#### Stimuli

On each trial, a 500-Hz tone was presented in white noise. The noise and the tone were generated separately in Praat (Boersma & Weenink, 2024) and mixed in MATLAB (The MathWorks Inc., 2022). All tones had a symmetrical in and out fade of 0.01 s. The noise and tone had a simultaneous onset and offset, and the total duration of each mixed audio file was the same as the duration of the object videos used in Experiments 2–4 (~4,250 ms). To mimic the object size change in Flavell et al. (2020), the tone changed briefly in pitch either early (1,900 ms) or late (2,883 ms) in a trial. The change in pitch was always to 550 Hz and lasted 200 ms. As well as these “change trials”, there were also “null trials” in which no pitch change occurred. To mimic the perceptual fluency manipulations (visual contrast/camouflage) used in Flavell et al. (2020), the SNR of the tone/noise mix was varied to make the tone easier or harder to detect. Four different SNRs were tested: 0 dB (easiest), ˗10 dB, ˗15 dB and ˗20 dB (hardest). The white noise was held constant at 0.1 Pa (~74 dB SPL) and the loudness of the tone adjusted to create the required SNRs. After mixing, all stimuli were RMS-equalised to 0.1 Pa.

#### Procedure

The experiment was run in Gorilla (www.gorilla.sc; Anwyl-Irvine et al., 2020). After giving informed consent, participants answered questions about their gender identity, age, and any hearing loss or hearing difficulties. They were also asked if they were wearing headphones. They were then asked to turn their volume down before listening to a calibration sound (white noise at 0.1 Pa) and turning their volume up until the sound was at a comfortable listening level. Next, they completed two brief audio checks. The first one checked that presented sounds were sufficiently audible. Participants heard three sets of digit triplets, spoken by a male talker of Standard Southern British English (SSBE). After each set, participants were asked to type the presented numbers into a text box. The second audio check determined whether or not autoplay was enabled on the participant’s browser, presented instructions about how to enable it if not, and gave participants the option to contact the researchers for further assistance if they were not able to activate it themselves.

Participants were familiarised with the change-detection task via four practice trials featuring only tones in isolation. They then heard an example of the white noise, before finally completing eight practice trials with the full tone-in-noise stimuli. These practice trials comprised a selection of the different SNRs (0 dB, ˗10 dB, ˗15 dB, ˗20 dB) and trial types (early change, late change, null) used in the main task. The main task comprised three early change trials, three late change trials, and one null trial at each of the four SNRs (28 trials in total). On each trial, participants were asked to press the spacebar when they detected a pitch change, or to make no response if no pitch change occurred. As in Flavell et al. (2020), feedback was given to indicate to participants whether they had responded correctly. The feedback took the form of the words “WELL DONE” or “INCORRECT”, presented on the screen for 1,000 ms after each trial. Responses were marked as correct if participants responded after the tone change and before the end of the audio file. Responses were marked as incorrect if participants responded outside of this time window, if they failed to respond to changes, or if they responded to null trials.

#### Analyses

Null trials and change trials were analysed separately. Individual change trials were excluded if the RT exceeded the mean + (2*SD) for each participant per SNR trial type, if the RT was less than 150 ms, or if the participant responded incorrectly (i.e., did not respond or responded outside of the time windows specified above). Criteria for participant exclusion were as follows: responding incorrectly to any of the audio check digit triplets; responding on more than 25% of null trials; having more than 25% of trials excluded according to the criteria above. No participants were excluded on these bases.

Linear mixed-effects models (LMMs) were used to model trial-level RTs for the change trials. One random by-participant intercept was included. The data did not support a more complex random-effects structure: models with an additional by-participant slope for SNR failed to converge. The fixed effect was SNR (0 dB, ˗10 dB, ˗15 dB, ˗20 dB). Likelihood ratio tests (LRTs) were used to determine whether the fixed effect of SNR contributed significantly to the model. Specifically, the full model as described above was compared to a reduced model that did not include SNR. All models used the BOBYQA optimiser (Powell, 2009) and a maximum of 10^9^ iterations. Post hoc pairwise comparisons for the LMMs were conducted using the *pairwise* argument from the *emmeans* function (Lenth, 2024), which conducted t-tests on the model data and produced Tukey-corrected *p*-values.

### Results

Accuracy was extremely high: no participants responded to any null trials, and only two of the 392 trials were excluded. Mean RTs (in ms) for each of the SNRs were as follows: 506.2 ± 135.5 (0 dB); 491.9 ± 139.3 (˗10 dB); 520.3 ± 137.7 (˗15 dB); 552.3 ± 158.7 (˗20 dB). The LMMs indicated that there was a significant main effect of SNR (*X*^2^(3) = 23.58, *p* <.001). Post hoc comparisons showed that RTs were significantly faster for the 0 dB and ˗10 dB conditions compared to the ˗20 dB condition (both *p* <.01). No other post hoc comparisons were significant.

### Discussion

RTs to the different SNR conditions differed as expected, with faster responses to the less perceptually challenging conditions (i.e., to the higher SNRs), and accuracy was extremely high throughout. This indicates a successful manipulation of perceptual fluency: participants were able to perceive the tones, and thus complete the task, in all conditions; however, the RTs indicate that the fluency with which they were able to do so was reduced as the SNRs became more challenging. Since the RT differences were relatively small overall, the most extreme SNRs (0 dB and −20 dB) were chosen for use in the subsequent experiments to ensure a sufficient difference between high and low fluency conditions.

## Experiment 2: Crossmodal transfer

Experiment 2 incorporated the auditory change-detection task described above into the object preference paradigm used by Flavell et al. (2020).

### Methods

#### Participants

No studies yet exist exploring crossmodal fluency-based preference effects, making estimations of likely power challenging. Furthermore, power calculations for LMMs are complicated by the need to estimate random variance/covariance parameters (Meteyard & Davies, 2020). However, Flavell et al. (2020) report very large effect sizes for their fluency-based preference effects (e.g., Experiment 2, effect of visual contrast: η^2^ =.414). These effect sizes are reported in the context of ANOVAs, and it is therefore not possible to estimate participant- or item-based random parameters; also, it is possible that effect sizes may be reduced when exploring effects crossmodally. Nevertheless, given the robust effects reported in Flavell et al. (2020) and the closely comparable visual stimuli used in the current study, we chose to use a similar sample size for our first experiment, the results from which were then used to guide more formal power analyses (see Experiment 3 below). Thirty-one volunteer participants (16 female, 13 male, one non-binary, one non-disclosed; mean age = 22.26 years (SD = 1.98)) were recruited as part of a Master’s project. A further nine participants (seven female, two male; mean age = 26.67 years (SD = 3.2)) were recruited via Prolific and reimbursed for their time, leading to a total sample size of 40, which is comparable to the sample sizes used in Flavell et al. (2020). All participants reported being under the age of 35 years, with no hearing loss or difficulties and with normal or corrected-to-normal vision.

#### Stimuli

The visual stimuli were based on the moving objects used in Flavell et al. (2020). On each trial, a video was presented in which an object moved in a straight line across a pale grey box in the centre of the screen. Thirty-six object videos were created for the current task. The objects were generated at random using bespoke MATLAB code with the following constraints: internal and external angles of ≥ 30°; minimum side lengths of ~13.5 mm; total areas between ~2,700 mm^2^ and ~5,401 mm^2^. Each object was presented as an outline of dark grey dots, each ~1.4 mm in diameter. The object’s starting position was a randomly chosen location around the perimeter of the box. On each trial, a fixation cross appeared for 500 ms, followed by a blank screen for 500 ms. The grey box then appeared containing the object in its starting position. The object remained stationary for 1,000 ms before moving in a straight line across the centre of the box over 3,000 ms at a constant velocity. The middle point of the object’s trajectory was always the centre of the box. The object halted at its final position for 250 ms before the box disappeared. There were also null video trials, in which the videos featured only a blank grey box and no object ever appeared.

As described above, the objects in Flavell et al. (2020) briefly changed size either early or late in their trajectory; furthermore, the objects were either harder (low contrast, high camouflage) or easier (high contrast, no/low camouflage) to detect, thus varying in perceptual fluency. In the current study, all visual objects were presented in the high-contrast/no-camouflage condition to maximise visual perceptual fluency, and no size changes occurred. Instead, the size change-detection task was replaced with the auditory pitch change-detection task. The auditory change-detection task was implemented as described in Experiment 1 above, except that only the most extreme SNRs (0 dB and ˗20 dB) were used.

On each trial, one tone-in-noise audio file and one object video were played simultaneously, with the onset/offset of the object video matched to the onset/offset of the audio file. There were 28 experimental trials. Of these, two were null video trials and four were null audio trials. Null videos and null audio were never paired together. Half of the trials with non-null audio used an SNR of 0 dB (more fluent) and half used an SNR of ˗20 dB (less fluent). Within each SNR, half of the trials featured an early pitch change and half featured a late pitch change. Participants were randomly allocated to one of two groups. The pairing of audio SNR and visual object was counterbalanced between the two groups, such that an object paired with a late-change 0 dB SNR audio file for Group 1 was paired with a late-change ˗20 dB SNR audio file for Group 2. This meant that the only variable manipulated within participants was the variable of interest (auditory perceptual fluency).

#### Procedure

The informed consent procedure, demographics questionnaire, and audio checks were as for Experiment 1. Participants were familiarised with the auditory change-detection task as follows: they first completed three trials featuring only tones in isolation; they then heard an example of the white noise, before completing three trials with the full tone-in-noise stimuli. These trials featured both selected SNRs (0 dB, ˗20 dB), an early change trial, a late change trial, and a null trial. Participants then completed four trials of the visual rating task featuring only object videos (i.e., no audio). Finally, participants completed six practice trials of the full task (auditory change-detection task plus subsequent object rating), before going on to complete the main experiment.

Participants were instructed to watch the object video while performing the auditory change-detection task. Once the video had ended, they were asked to rate each object for liking. They were presented with a Likert scale in the form of a horizontal line and asked to move the central marker towards the right if they liked the object or towards the left if they didn’t, with how far left or right indicating the extent of their (dis)like. The line was presented to participants without demarcations; however, the lower and upper limits corresponded to arbitrary values of −100 and +100, respectively. On all trials, there was also a “NO SHAPE” button on the screen, which participants could click in response to null video trials. As in Experiment 1, feedback was given on performance in the auditory task for both the practice and the experimental trials.

#### Analyses

Exclusion criteria for individual trials and participants were as for Experiment 1, with the added criterion of failing to select the “NO SHAPE” option for more than 25% of null video trials.

Two LMMs were used for the main analysis: one to model trial-level RTs and one to model trial-level object ratings. In both cases, only audio change trials paired with non-null videos were analysed. For the RT model, the random-effects structure included an intercept for participant and a by-participant slope for SNR. For the object ratings model, the random-effects structure included an additional intercept for object (the model did not converge with a by-object slope for SNR). In both cases, the sole fixed effect was SNR (0 dB, ˗20 dB). This was treatment coded with −20 dB as the reference level. Likelihood ratio tests (LRTs) were used as in Experiment 1 to determine whether the fixed effect of SNR contributed significantly to the model. All other parameters were as for Experiment 1.

### Results

**Fig. 1:**
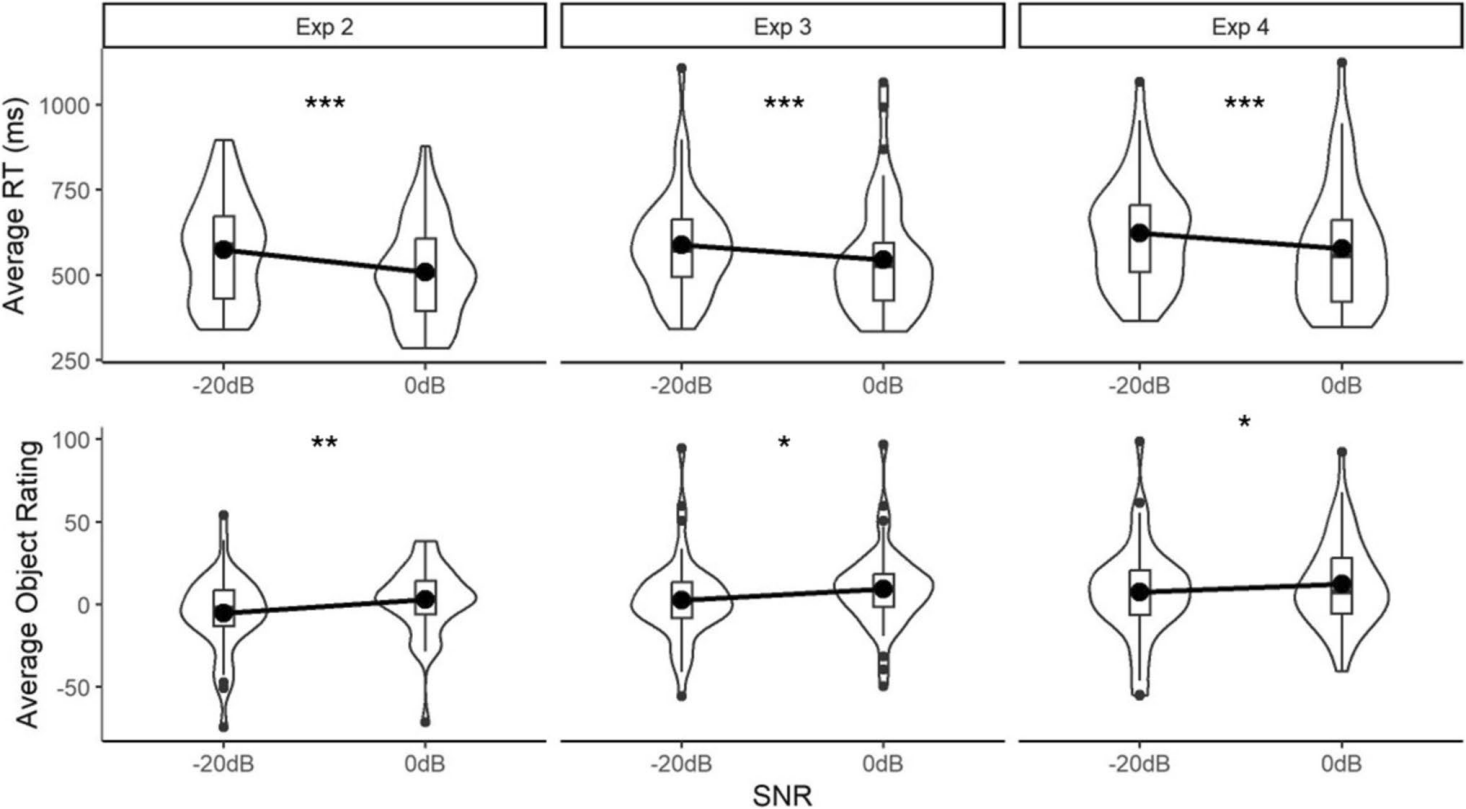
Average reaction times (RTs) in the auditory change-detection task (**top panel**) and ratings for the visual objects (**bottom panel**), for the fluent (0 dB) and less fluent (˗20 dB) signal-to-noise ratio (SNR) conditions in Experiments 2, 3 and 4. For the boxplots within the violins, lower and upper hinges of boxes correspond to the first and third quartiles, and whiskers extend to the largest and smallest values no further than 1.5*IQR (interquartile ratio) from the mean. Values further than 1.5*IQR from the mean are plotted as outliers. Horizontal bars indicate condition medians and large black dots indicate condition means. The violins themselves represent the data distribution, with the width of the violin at any point indicating the density of data points at that value. Significance tests refer to the generalised linear mixed models (GLMMs) reported in the main text (*** = *p* <.001, ** = *p* <.01, * = *p* <.05)

Of the 880 audio change trials, 24 were excluded. Mean RTs and object ratings for the two SNRs are shown in Fig. 1. The LRT for the RT model indicated that there was a significant main effect of SNR (*X*^2^(1) = 21.91, *p* <.001; coefficient = −63.48; SE = 11.80), reflecting the fact that RTs were faster for the more fluent (less challenging) SNR (0 dB). The LRT for the object-rating model indicated that there was a significant main effect of SNR (*X*^2^(1) = 7.01, *p* <.01; coefficient = 8.46, SE = 3.05), with higher object ratings for the more fluent SNR.

### Discussion

The results suggest that perceptual fluency-based preference effects can transfer crossmodally from the auditory to the visual domain: visual objects presented concurrently with perceptually fluent auditory stimuli received higher liking ratings than the same visual objects presented with less perceptually fluent auditory stimuli.

Although the RT difference between the fluent and less fluent auditory conditions was relatively small (~63 ms), it was at least comparable to, and in some cases larger than, the RT differences obtained by Flavell et al. (2020) for the comparable conditions of their visual manipulation (high vs. low contrast; no camouflage). The object ratings overlapped zero, indicating that the auditory fluency manipulation created both dislike (negative values) and liking (positive values) for the objects, hence altering the valence of participants’ preferences as well as simply the magnitude.

In conclusion, the results point to the existence of the crossmodal transfer of fluency-based preference effects in the audiovisual domain. However, since this is the first time that such an effect has been demonstrated, we chose to re-run Experiment 2 to check that the result replicated.

## Experiment 3: Replication

### Methods

#### Participants

In order to ensure that the replication was sufficiently powered, the R package *simr* was used as detailed in Kumle et al. (2021; see also https://lkumle.github.io/power_notebooks/Scenario1_notebook.html) to extend the existing dataset from Experiment 2 and explore power for different sample sizes given a similar model structure (LMM) and significance testing procedure (LRTs). It was determined from theses simulations that, although the effect of SNR on RT was robust for a range of sample sizes, the effect of SNR on object ratings only exceeded 80% power (at an alpha of 0.05) for a sample size of 50 or more participants. Fifty participants were therefore recruited via Prolific using the same inclusion criteria as for the previous experiments (28 female, 21 male; mean age = 29.04 years (SD = 3.98); no data available for one participant).

The stimuli and procedure were identical to those in Experiment 2.

#### Analyses

Exclusion criteria for individual trials and participants were as for Experiment 2. Two LMMs were again used for the main analysis and restricted to the audio change/non-null video trials, as before. For the RT model, the random-effects structure included an intercept for participant and a by-participant slope for SNR. For the ratings model, the random-effects structure included intercepts for participant and object, and by-participant and by-object slopes for SNR. In both cases, the sole fixed effect was SNR (0 dB, ˗20 dB). Significance testing and all other parameters were as for Experiment 2.

### Results

In total, 25 out of the 1,100 audio change trials were removed. Mean RTs and object ratings for each of the SNRs are shown in Fig. 1. The LRT for the RT model indicated that there was a significant main effect of SNR (*X*^2^(1) = 16.83, *p* <.001; coefficient = −43.11, SE = 9.64), reflecting the fact that RTs were faster for the more fluent (less challenging) SNR (0 dB). The LRT for the ratings model indicated that there was a significant main effect of SNR (*X*^2^(1) = 5.03, *p* <.05; coefficient = 6.56, SE = 2.78), reflecting the fact that object ratings were higher for the more fluent SNR.

## Discussion

The results from Experiment 3 replicate those from Experiment 2: objects presented concurrently with more perceptually fluent auditory stimuli received higher liking ratings than the same objects presented with less perceptually fluent auditory stimuli. The results again support the hypothesis that perceptual fluency-based preference effects can transfer crossmodally from the auditory to the visual domain.

The magnitude of the RT difference was similar to that observed in the previous experiments. Unlike Experiment 2, the mean object ratings for the two auditory fluency conditions were both within the range of positive values, suggesting that – in this study – auditory fluency influenced only the magnitude and not the valence of participant preferences.

Having replicated our original finding of crossmodal transfer of auditory processing fluency to visual object preference, we now turn to the question of how such crossmodal transfer might occur. As discussed in the *Introduction*, it is possible that transfer occurs via a global effect, in which positive affect generated by fluent processing of one stimulus spreads to surrounding stimuli in other domains. This global effect is similar to a crossmodal “halo effect”, in which a range of judgements are influenced by a single characteristic (e.g., Nisbett & Wilson, 1977). This effect can be thought of as a form of crossmodal *integration*, in which information is combined across sensory modalities during perceptual judgements (Bizley et al., 2016). In this scenario, there is not necessarily any perceived link between the different channels of information; rather, the positive judgements associated with one channel (in this case, auditory fluency) are – consciously or otherwise – taken into account when forming judgements of the information in another channel (the visual objects). Alternatively, transfer may instead be due not to generalised integration but to an object-specific effect, in which stimuli in different domains are perceptually bound into a single multisensory object, any aspect of which is preferred if one of its constituent parts is more fluently processed. This object-specific effect is akin to an effect which Bizley et al. refer to as crossmodal *binding*, a special case of integration in which perceptual features across modalities are grouped into a single unified multisensory object (*ibid.*). In this scenario, the two stimuli are perceived as deriving from the same source (e.g., the object is perceived as producing the sound). This would be in line with the so-called “unity assumption” – the tendency to assume that multiple cues derive from the same object or event – which has been proposed as a key mechanism underlying our ability to bind (or segregate) multimodal features of the same (or different) object(s) (Chen & Spence, 2017). Relevant here is the fact that the unity assumption has been shown to be more likely when there is close temporal correspondence between auditory and visual stimuli (see Spence, 2007, for a review). This in turns means that, if binding is necessary for crossmodal transfer of preference effects to take place, then such transfer is unlikely to occur in situations without close crossmodal temporal correspondence.

Experiment 4 was designed to exploit the temporal-correspondence feature in order to adjudicate between integration and binding as possible mechanisms for the crossmodal transfer of fluency-based preference effects observed in the previous experiments. Since binding (but not integration) appears to rely on relatively close temporal correspondences, a temporal lag was introduced between the onset of the visual and auditory stimuli. Such a lag should weaken or negate crossmodal binding but still allow for crossmodal integration. Therefore, if we continue to observe similar crossmodal transfer of fluency-based preference effects in the presence of the lag, we can conclude that crossmodal transfer relies primarily on integration. However, if crossmodal transfer is no longer observed, we can conclude that it requires binding.

## Experiment 4: Temporal lag

Experiment 4 was similar to Experiments 2 and 3, but included a temporal lag between the onsets of the visual and auditory stimuli.

### Methods

#### Participants

Experiment 4 was designed to adjudicate between two possible explanations for the results obtained in Experiments 2 and 3. We were therefore only interested in detecting effect sizes similar to (or larger than) those obtained in Experiment 2, and so used the sample size indicated by our previous power analysis. Fifty participants were recruited and tested using the same procedure and exclusion criteria as in Experiments 2 and 3 (22 female, 28 male; mean age = 29.40 years (SD = 4.33)).

#### Stimuli

The same counterbalanced pairings of auditory stimuli and object videos were used as in Experiments 2 and 3. However, a temporal lag was introduced between the appearance of the objects and the onset of the auditory stimuli, such that the audio preceded the object for half of the trials and followed it for the other half.

The duration of the temporal lag was determined by several criteria. First, to preclude the possibility of binding as far as possible, the onsets of the two components needed to fall outside the recognized duration of the temporal binding window (TBW; Stevenson & Wallace, 2013). The TBW has been measured with a wide variety of stimuli, tasks and criteria (see Vatakis & Spence, 2010), making definitive assessments of its size for any given combination of audiovisual objects challenging. It is also typically found to be asymmetric for audiovisual stimuli, with larger values when video leads audio. Behavioural studies suggest that asynchrony can be detected with a lag as short as ~200 ms, and reliably detected in either direction with a lag of ~500 ms (Conrey & Pisoni, 2006; Miller & D’Esposito, 2005; Stevenson & Wallace, 2013). To ensure that the lag not only could be detected, but also actively hindered binding, this value was doubled to 1,000 ms. However, as described in the *Methods* for Experiment 2, objects in the videos remained stationary for 1,000 ms before starting their trajectory across the screen. To avoid the audio onset coinciding with the onset of movement in video-lead trials (which could itself have created the perception of temporal coherence) the lag was lengthened to 1,200 ms. This was judged to create a suitable degree of non-coherence between the audio onset and appearance of the object, and between the audio onset and the onset of object motion.

For audio-lead trials, an empty grey box appeared on-screen at the point of the audio onset; the video itself began (i.e., the object first appeared) 1,200ms later. For video-lead trials, the audio began 1,200 ms after the onset of the video, and an empty grey box remained on-screen for 1,200 ms after the video ended (i.e., after the object disappeared). Null audio and video trials were implemented as in Experiments 2 and 4.

#### Procedure

The same procedure was used as for Experiment 2. The six practice trials for the full task included three audio-lead and three video-lead trials, and the feedback given to participants reflected whether or not they had responded in the appropriate time window relative to the auditory pitch change, regardless of whether or not the auditory stimulus as a whole preceded or followed the object video.

#### Analyses

The exclusion criteria for individual trials and participants were the same as those used in Experiments 2 and 3. Two LMMs were used for the main analysis, again restricted to the audio change/non-null video trials. For the RT model, the random-effects structure included an intercept for participant and a by-participant slope for SNR. For the ratings model, the random-effects structure included intercepts for participant and object, and by-participant and by-object slopes for SNR. In both cases, the sole fixed effect was SNR (0 dB, ˗20 dB). Significance testing and all other parameters were as for Experiments 2–4.

Two additional exploratory LMMs were run to examine potential differences in RTs and/or object ratings across the two lag types (audio-lead vs. video-lead). For both the RT-lag and ratings-lag models, the random-effects structure included an intercept for participant and by-participant slopes for SNR and lag type. The data did not support a more complex random-effects structure: models with a by-participant slope for SNR × lag type failed to converge. In both models, the fixed effects were SNR (treatment coded as above), lag type (treatment coded with audio-lead as the reference level), and SNR × lag type. Likelihood ratio tests (LRTs) were used as before to determine which fixed effects contributed significantly to the model.

### Results

In total, 47 of the 1,100 audio change trials were removed. Mean RTs and shape ratings for each of the SNRs are shown in Fig. 1. The LRT for the RT model indicated that there was a significant main effect of SNR (*X*^2^(1) = 13.43, *p* <.001; coefficient = −46.03, SE = 11.52), reflecting the fact that RTs were faster for the more fluent (less challenging) SNR (0 dB). The LRT for the ratings model revealed a significant main effect of SNR (*X*^2^(1) = 4.43, *p* <.05; coefficient = 4.98, SE = 2.26), with higher object ratings for the more fluent SNR.

Results from the RT-lag model indicated a significant main effect of SNR as expected (*X*^2^ (1) = 5.089, *p* = <.05; coefficient = −37.44, SE = 16.36). There was also a significant main effect of lag type, reflecting slower RTs to audio-lead trials (*X*^2^ (1) = 11.111, *p* <.001; coefficient = −54.80, SE = 16.10). The SNR × lag type interaction was not significant (*p* =.45).

Results from the ratings-lag model indicated a significant main effect of lag type (*X*^2^ (1) = 5.284, *p* <.05; coefficient = −7.06, SE = 3.05), reflecting higher liking ratings for audio-lead trials. However, the main effect of SNR did not reach significance in this model (*p* = 0.15). There was also no significant SNR × lag type interaction (*p* = 0.86).

### Discussion

The main results from Experiment 4 mirror those of Experiments 2 and 3: visual objects presented concurrently with more perceptually fluent auditory stimuli received higher liking ratings than the same objects presented with less perceptually fluent auditory stimuli. The magnitude of the RT difference was similar to that observed in the previous experiments. As in Experiment 3, but unlike Experiment 2, the mean object ratings for the two auditory fluency conditions were both positive, suggesting that auditory fluency was influencing the magnitude but not the valence of participant preferences.

These results suggest that the crossmodal transfer of fluency-based preference effects persists even when the temporal correspondence between stimuli in the different modalities is disrupted. This in turn suggests that crossmodal transfer is likely to be occurring due to crossmodal integration (i.e., the use of ostensibly irrelevant information from other modalities during judgement-making about an object in a target modality) rather than crossmodal binding (i.e., the grouping of features from different modalities into a single, unified multisensory object).

It is possible that the temporal lag introduced in the current experiment was simply too small to fully disrupt crossmodal binding processes: there was an overlap between audio and video of ~3,050 ms (the majority of the stimulus presentation window), which could be sufficient for crossmodal binding to take place if it is going to occur (see e.g., Stevenson & Wallace, 2013). Furthermore, although every effort was made to select a lag duration that placed the auditory and visual stimuli well outside of the TBW, the heterogeneity of the existing literature on the TBW makes it difficult to estimate its size for any specific combination of stimuli and task. Nevertheless, the chosen lag of 1,200 ms was considerably larger than existing estimates of the TBW; it ensured a clear separation between the onsets and offsets of the audio and video components of the task, and there were no other potential points of coincidence (e.g., the audio onset did not coincide with the start of the object’s movement).

Future studies could systematically vary the extent of the overlap between the auditory and visual components in order to determine the degree of temporal co-existence required for crossmodal transfer of fluency-based preference effects to take place. If crossmodal transfer is indeed due to some kind of globalised positive affect, then it is possible that this may endure for a period after the (dis)fluent processing has occurred in one stimulus modality, affecting judgements for objects presented in a different modality for some time after the offset of the (dis)fluent stimulus. This, however, remains an empirical question.

We also found an effect of lag type (audio-lead vs. video-lead) on both RTs and object ratings. It is worth observing that effects of lag type were not the focus of this experiment, and the models we ran to assess these were exploratory, since we were not powered to investigate this question. However, two things are notable: first, RTs to audio-lead trials were slower. We attribute this to distraction caused by the onset of the video, which – although it did not coincide with the pitch change – always occurred part-way through an ongoing audio trial. It seems likely that this captured participants’ attention and thus slowed their responses to the audio stimuli. Second, objects presented with audio-lead trials were rated more positively. We suggest that this may be related to the timeline of the two trial types. On audio-lead trials, the objects were visible immediately before participants provided their liking rating. For video-lead trials, however, there was a short gap before the ratings were obtained during which the object had disappeared and only the audio stimulus was being presented; on these trials, participants therefore needed to recall the object before rating it. The cognitive effort associated with this recall process may have reduced the overall experience of fluency, thus leading to lower liking ratings for these objects.

As discussed, these conclusions regarding the effects of lag type must be treated with caution given the exploratory nature of the analyses. Indeed, in the ratings-lag model, the main effect of SNR did not reach significance, an effect which we attribute to lack of power. At the very least, however, we believe that the results from these models suggest that participants were engaging with the visual objects rather than simply responding according to their feelings about the auditory trials. The possible distraction caused by video onset, coupled with higher liking ratings when participants had seen the object more recently, suggest that they were indeed attending to the objects and rating them as instructed. Furthermore, the fact that audio-lead trials produced slower responses than video-lead trials, but were nevertheless judged more favourably, also suggests that slower responses to the auditory stimuli per se did not change participants’ liking ratings – rather, ratings were only affected by RT changes linked to perceptual fluency, thus supporting a fluency-based interpretation of the data.

The results so far appear to point to the existence of crossmodal transfer of fluency-based preference effects in the audiovisual domain. However, it is worth observing that the object-in-motion paradigm employed in Flavell et al. (2020) and extended here is relatively unusual: the majority of studies exploring unimodal (visual) perceptual fluency-based preference effects have used static images as the to-be-rated stimuli. In the current crossmodal context, the use of objects in motion seems appropriate, in as much as auditory stimuli unfold over time. Whether similar crossmodal transfer effects would be observed with static images is unclear. On the one hand, the results from Experiment 4 suggest that the positive affect generated by fluent processing of one stimulus may generalise to other stimuli regardless of any perceived temporal alignment between them. On the other hand, it is possible that there is something unique about objects in motion that facilitates the crossmodal transfer of fluency effects, either by increasing the likelihood of transfer and/or by strengthening any transfer that occurs. For example, objects in motion may be more perceptually vivid than static objects. Vividness can be defined as an experience of physical/temporal proximity or emotional appeal, and more vivid information is typically more interesting, more emotionally arousing, and enhances mental involvement (Nisbett & Ross, 1980). If objects in motion are more vivid than static objects, this may increase the likelihood of perceptual fluency influencing preference judgements (via increased mental involvement) and/or may increase the strength of any positive/negative fluency effects (via higher emotional arousal). It is also possible that objects in motion more strongly invite the attribution of sentience than static objects. In a classic study by Heider and Simmel (1944), when participants were asked to interpret a video involving moving geometric shapes, they did so in terms of intentions and social interactions. If participants in the current study attributed any degree of sentience to the objects as a result of the motion, this may have increased their engagement with the task and/or their ability to (dis)like the objects – hence increasing the likelihood of fluency-based preference effects and/or strengthening any such effects.

Some evidence to support this view can be found in Roggeveen et al. (2015). In this study, participants saw objects that varied in their hedonic or utilitarian value (i.e., their ability to provide pleasurable vs. functional/practical outcomes). The objects were presented in both dynamic (i.e., object-in-motion) and static conditions. Dynamic presentation was found to increase preference for hedonically superior objects but had no effect for objects that were superior in utilitarian aspects. In other words, showing objects in motion appeared to enhance effects specifically related to enjoyment and liking.

To explore this possibility, Experiment 5 therefore replicated Experiments 2 and 3 using static objects instead of objects in motion.

## Experiment 5: Static objects

Experiment 5 was identical to Experiment 2, except that each trial used a static image of the relevant object from Experiment 2 instead of a video of the object in motion.

### Methods

#### Participants

In line with the rationale outlined above, fifty-one eligible participants were recruited and reimbursed via Prolific and tested via Gorilla. However, to ensure equal numbers of participants for each counterbalanced pairing of auditory trials and objects, the final participant to take part was excluded from further analyses. Fifty participants (19 female, 28 male, three non-binary; mean age = 29.82 years (SD = 4.27)) were therefore included in the final analyses, with the same inclusion criteria as for Experiment 2.

#### Stimuli

For each object video used in Experiment 2, one frame was extracted corresponding to the moment at which the object was at the centre of the grey box. On each trial, the relevant frame was presented in place of its object video. Each frame remained on-screen for the same overall duration as the object videos. Null audio and image trials, and feedback, were implemented as above.

#### Procedure

The procedure was identical to Experiment 2, with the exception that all object videos in practice trials and experimental trials were replaced with their respective static frame.

#### Analyses

Exclusion criteria for individual trials and participants were as for Experiment 2. Two LMMs were again used for the main analysis: one to model trial-level RTs and one to model trial-level object ratings. Only audio change trials paired with non-null videos were analysed. For the RT model, the random-effects structure included an intercept for participant and a by-participant slope for SNR. For the ratings model, the random-effects structure included intercepts for participant and object, and by-participant and by-object slopes for SNR. In both cases, the sole fixed effect was SNR (0 dB, ˗20 dB). Significance testing and all other parameters were as for Experiment 2.

### Results

**Fig. 2:**
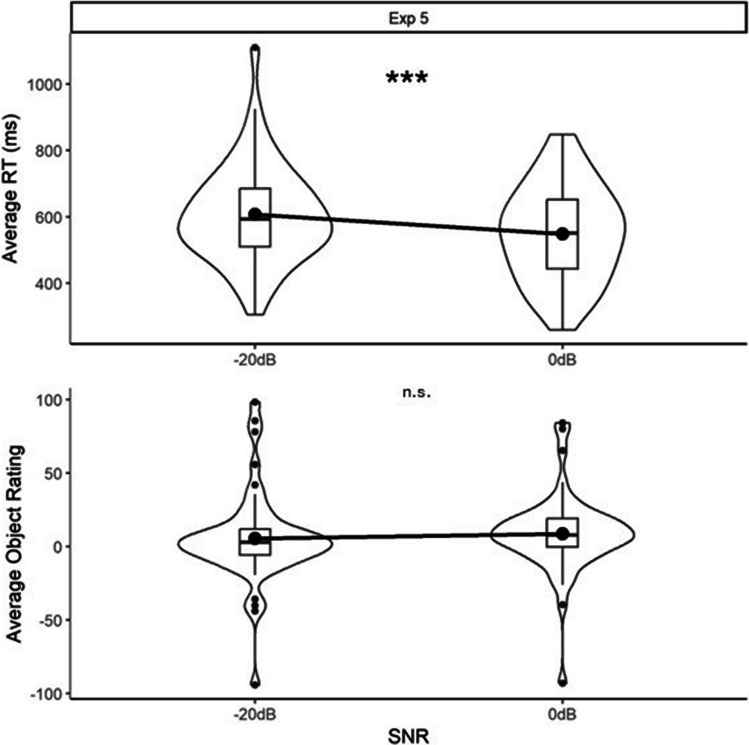
Average reaction times (RTs) in the auditory change-detection task (**top panel**) and ratings for the visual objects (**bottom panel**), for the fluent (0 dB) and less fluent (˗20 dB) signal-to-noise ratio (SNR) conditions in Experiment 5. The specifications for the boxplots and violins are as for Fig. 1. Significance tests refer to the generalised linear mixed models (GLMMs) reported in the main text (*** = *p* <.001, n.s. = *p >*.05)

In total, 53 of the 880 audio change trials were removed. Mean RTs and object ratings for each of the SNRs are shown in Fig. 2. The LRT for the RT model indicated that there was a significant main effect of SNR (*X*^2^(1) = 21.95, *p* <.001), with faster RTs for the more fluent SNR (0 dB) than the less fluent SNR (˗20 dB). The LRT for the object ratings model indicated no significant effect of SNR (*X*^2^(1) = 1.90, *p* =.17).

Since the object ratings model did not show the expected effect of SNR, a follow-up LMM was run to directly compare the object ratings from the original moving-object experiments (Experiments 2 and 3) to the object ratings from the current (static-object) experiment (Experiment 5). This model had the same random-effects structure as the object ratings model from the current experiment and included fixed effects of SNR, Motion (moving vs. static), and an interaction of SNR × Motion, with the interaction term being the term of interest. An LRT comparing this model to a model without the interaction term showed no significant interaction between SNR and Motion (*X*^2^(1) = 1.96, *p* =.16). Thus, although the effect of SNR on object ratings reached significance in Experiments 2 and 3, but not in Experiment 5, there was no meaningful difference between the effect of SNR in the two experiments.

#### Discussion

The results again confirmed the success of the perceptual fluency manipulation: RTs for the fluent (0 dB SNR) auditory condition were significantly faster than for the less fluent (−20 dB SNR) condition. The magnitude of the RT difference was similar to that of Experiment 2. Unlike in Experiments 2 and 3, there was no significant effect of auditory fluency on object ratings. However, a direct comparison between the current experiment and Experiments 2 and 3 showed no difference between the effect of SNR on object ratings across the two types of experiment (static vs. dynamic objects), making this apparent disparity difficult to interpret. To more directly explore the moving-versus-static contrast, it is necessary to compare ratings for moving versus static objects within the same participant sample. We therefore conducted Experiment 6, which combined moving and static objects within the same experimental task.

## Experiment 6: Static and moving objects

### Methods

#### Participants

To attempt to ensure sufficient power, we recruited twice the sample size used in Experiments 2–5. One hundred participants were recruited via Prolific using the same inclusion criteria as for the previous experiments (63 female, 37 male; mean age = 28.7 years (SD = 4.61)).

The stimuli and procedure were identical to those in Experiment 2, with the exception that each participant was presented with half of the visual objects as videos (as in Experiments 2, 3 and 4) and the other half as static images (as in Experiment 5). There were 28 experimental trials as before. Of these, two were null video trials and four were null audio trials. Null videos and null audio were never paired together. Half of the trials with non-null audio used an SNR of 0 dB (more fluent) and half used an SNR of ˗20 dB (less fluent). Within each SNR, half of the trials featured moving objects (i.e., videos) and half featured static images of objects. Also within each SNR, half of the trials featured an early pitch change and half featured a late pitch change. Participants were randomly allocated to one of four groups. The pairing of audio SNR, visual object, and trial type (static vs. moving) was counterbalanced between the groups, such that a moving object paired with a late-change 0 dB SNR audio file for Group 1 was paired with a late-change ˗20 dB SNR audio file for Group 2, and the same object was paired as a static image with a late-change 0 dB SNR audio file for Group 3 and a late-change ˗20 dB SNR audio file for Group 4. This meant that the variables manipulated within participants were the two variables of interest (auditory perceptual fluency and trial type).

#### Analyses

Exclusion criteria for individual trials and participants were as for Experiment 2. We first ran two LMMs as used in the previous experiments, i.e., one to model trial-level RTs and one to model trial-level object ratings, using only audio change trials paired with non-null videos and with the sole fixed effect of SNR (0 dB, ˗20 dB). For the RT model, the random-effects structure included an intercept for participant and a by-participant slope for SNR. For the ratings model, the random-effects structure included intercepts for participant and object (defined as the object identity, regardless of whether it was moving or static), and by-participant and by-object slopes for SNR. All other parameters were as for Experiment 2.

We then ran two further LMMs on the same trials which included fixed effects of SNR, Motion (moving vs. static), and their interaction. For the RT-interaction model, we initially attempted to fit a maximal random-effects structure that included an intercept for participant and a by-participant slope for the interaction of SNR and Motion. However, the most complex structure that the data would support was an intercept for participant and a by-participant slope for SNR. For the ratings-interaction model, the data supported intercepts for participant and object, and by-participant and by-object slopes for both SNR and Motion, although not their interaction. Significance testing used LRTs, as for the previous experiments. Specifically, the full model as described above was compared to a reduced model that did not include the effect of interest (SNR, Motion or SNR × Motion).

### Results

**Fig. 3:**
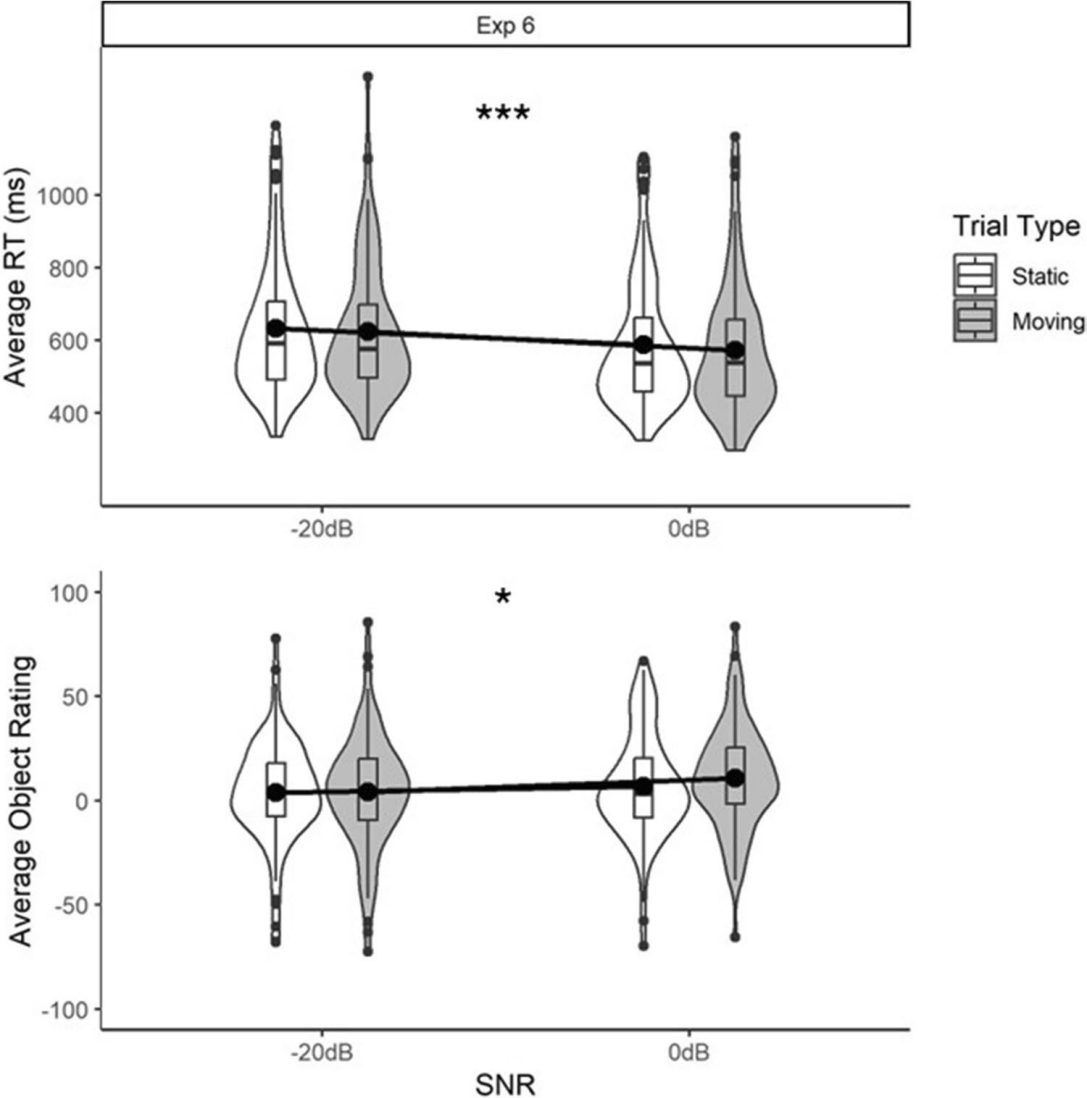
Average reaction times (RTs) in the auditory change-detection task (**top panel**) and ratings for the visual objects (**bottom panel**), for the fluent (0 dB) and less fluent (˗20 dB) signal-to-noise (SNR) conditions and moving vs. static objects in Experiment 6. The specifications for the boxplots and violins are as for Fig. 1. Significance tests refer to the generalised linear mixed models (GLMMs) reported in the main text (*** = *p* <.001, * = *p <*.05)

In total, 68 out of the 2,200 audio change trials were removed. Mean RTs and object ratings for each of the SNRs are shown in Fig. 3. The LRT for the RT model indicated that there was a significant main effect of SNR (*X*^2^(1) = 22.77, *p* <.001), reflecting the fact that RTs were faster for the more fluent (less challenging) SNR (0 dB). The LRT for the ratings model indicated that there was a significant main effect of SNR (*X*^2^(1) = 5.16, *p* <.05), reflecting the fact that object ratings were higher for the more fluent SNR.

The LRTs for the RT-interaction model again indicated a significant main effect of SNR (*X*^2^(1) = 13.85, *p* <.001). There was no significant effect of Motion and no significant SNR × Motion interaction (both *p* > 0.3). The LRTs for the ratings model showed no significant effects of SNR, Motion, or SNR × Motion (all *p*s > 0.1).

### Discussion

The overall results from Experiment 6 replicate those from Experiments 2, 3 and 4: objects presented concurrently with more perceptually fluent auditory stimuli received higher liking judgements than the same objects presented with less perceptually fluent auditory stimuli. Thus, when considered independent of the mode of object presentation (moving vs. static), the results again support the hypothesis that perceptual fluency-based preference effects can transfer crossmodally from the auditory to the visual domain.

Adding motion to the RT model indicated no effect of the mode of object presentation on responses to the auditory task. This suggests that participants were not, for example, more distracted from the auditory task by the moving than static objects in either or both fluency conditions. When motion was added to the model of object ratings, there was no significant interaction between SNR and motion, despite the numerical trend for the moving objects to produce larger differences between the two auditory fluency conditions. In other words, there was no statistical evidence that the effect of auditory fluency was weaker for the static than the moving objects. However, this model also showed no main effect of auditory fluency, in contrast to the overall results.

Taken together, these results again confirm the existence of overall fluency-based preference effects, but suggest that the effect is sufficiently small for even one additional predictor (i.e., motion) to be able to render it statistically undetectable. It is therefore tempting to conclude that the numerically smaller difference between the two auditory fluency conditions for static objects was driving the lack of an overall fluency effect in the more complex model, implying a real but undetectable difference between fluency-based preference effects for static versus moving objects. However, this is not supported statistically, and we must assume for now that there are no meaningful differences in the crossmodal transfer of fluency-based preference effects for moving versus static objects. The question nevertheless merits further exploration in future studies.

## General discussion

Perceptual fluency-based preference effects are well established unimodally. When a visual or auditory object is more easily processed – due to familiarity, exposure duration, signal-to-noise ratio or some other perceptual characteristic – it tends to receive higher liking ratings. However, it is unclear whether and how such fluency-based preference effects operate crossmodally. Specifically, it is unknown whether a more perceptually fluent stimulus presented in one modality can affect preference for an unrelated object presented in another modality.

We addressed this question in a series of experiments which extended the unimodal paradigm used by Flavell et al. (2020) into the audiovisual domain. In Flavell et al.’s original paradigm, participants were asked to detect a brief size change in a moving object, after which they rated their liking of the object. The objects were made easier or harder to detect (i.e., more or less fluent perceptual processing) by manipulating contrast and/or camouflage. The results showed higher liking ratings for more fluently processed objects.

In the current study, participants saw similar objects, but there were no size changes and all the objects were maximally perceptually fluent (i.e., high contrast, no camouflage). The size change task was replaced by an auditory change task presented simultaneously with the object. In this task, participants had to detect a brief pitch change in a pure tone masked by white noise. The fluency of the auditory task was manipulated by changing the SNR, such that the tone was easier or harder to detect. The success of this fluency manipulation was confirmed by Experiment 1, in which RTs were significantly faster at higher (easier) compared to lower (harder) SNRs.

A significant difference in RTs to a higher versus lower SNR was observed in all the experiments, indicating that the desired difference in auditory perceptual fluency was present throughout. Critically, in Experiments 2, 3, 4 and 6, objects presented concurrently with more perceptually fluent auditory stimuli (higher SNR) received higher liking judgements than the same objects presented with less perceptually fluent auditory stimuli (lower SNR). This therefore suggests that perceptual fluency-based preference effects can transfer crossmodally from the auditory to the visual domain.

In Experiment 4, as well as providing a further replication of the fluency effect, we also considered potential underlying mechanisms. In particular, we attempted to disambiguate crossmodal integration versus crossmodal binding. Crossmodal integration refers to a process in which information is combined across sensory modalities during perceptual judgements (Bizley et al., 2016). In the current studies, this would mean that the positive affect associated with more fluent auditory trials is – consciously or otherwise – taken into account when forming judgements of the visual objects. In other words, fluency-based effects from one stimulus “spread” to concurrent stimuli in other domains, but without participants necessarily perceiving any causal link or other relationship between the stimuli in question. However, for crossmodal transfer of fluency-based preference effects to take place, it may be necessary for there to be crossmodal binding. This refers to a special case of integration in which perceptual features across modalities are grouping into a single unified multisensory object (*ibid.*), any aspect of which may be preferred if one of its constituent parts is more fluently processed. The binding of stimuli in different domains into a single multisensory object relies on close temporal correspondence between those stimuli (Spence, 2007), a feature we exploited in Experiment 4 in an attempt to disambiguate between these two possible mechanisms. By introducing a lag between the onsets of the audio and video stimuli, we disrupted the audiovisual temporal correspondence. However, crossmodal preference effects were still observed, suggesting that these effects might be driven by a global mechanism more akin to crossmodal integration than binding.

The results also indicate that this was a “true” crossmodal effect, rather than a situation in which participants were – despite the task instructions – largely ignoring the visual stimuli and primarily responding according to their feelings about the auditory task. As discussed in the Experiment 4 Discussion, the observed effects of lag type point towards a situation in which participants were paying attention to the visual objects and providing ratings specifically in response to what they had seen. Also, we observed a considerable range of liking ratings, despite the highly homogenous nature of the auditory stimuli. As discussed with regard to the results from Experiment 2, the auditory stimuli took one of only a very limited number of forms repeated throughout. It seems implausible that participants could have generated such a range of liking ratings for the visual objects if their responses were driven only by their feelings about the auditory task. Nevertheless, future studies should investigate this possibility by exploring whether apparently crossmodally-induced changes in preference for visual objects endure beyond the immediate auditory environment of their presentation and/or whether preference judgements translate into active behaviours, such as choosing one object over another for later use. Such effects have already been demonstrated in the unimodal context (e.g., McKean et al., 2020).

Significant audiovisual crossmodal preference effects were only observed for visual objects in motion: the same effects were not observed when the to-be-judged objects were static images (Experiment 5). However, a comparison of the effect of SNR on object ratings for the video versus image experiments showed no significant cross-experiment differences; and a further follow-up experiment (Experiment 6), comparing judgements of static and moving objects within the same study and participants, also showed no significant interaction between object motion and SNR. It may be the case that the use of static objects simply weakened, rather than removed, the crossmodal transfer of fluency-based preference effects. However, this was not supported statistically. In short, this result is difficult to interpret and requires further exploration.

The dual-task nature of our paradigm invites consideration of the potential role of perceptual load in our experiments. Lavie’s perceptual load theory of attention proposes that the effect of distractor stimuli is smaller under conditions of high perceptual load than low perceptual load (e.g., Lavie et al., 2014). Under high perceptual load, processing capacity is fully occupied by the to-be-attended information, meaning that unattended information is ignored. Under low perceptual load, however, there is spare processing capacity, which is automatically allocated to task-irrelevant information regardless of the perceiver’s intentions. Effects of load may have operated crossmodally in our study, such that higher processing load in one domain (the less fluent auditory condition) reduced processing of task-irrelevant information in a different domain (visual objects). However, this does not convincingly account for the results presented here, for several reasons. First, there is considerable debate as to whether or not perceptual load theory is applicable crossmodally (see Sandhu & Dyson, 2016, for a review). Second, although the videos were irrelevant with respect to the auditory domain (i.e., they provided no information relevant to the tone-in-noise task), they were not strictly task-irrelevant, since participants knew that they would have to rate the objects at a later stage. Third, even if the higher perceptual load generated by the less fluent auditory trials reduced participants’ ability to sustain their attention to the visual objects, there is no reason to suppose that this would necessarily lead to lower ratings for those objects. It is also important to remember that a systematic drop in object ratings in the less fluent auditory condition caused by negative affect associated with auditory effort would itself represent a crossmodal transfer of fluency-based preference effects, as argued for here. Nevertheless, future studies should attempt to rule out load effects per se as a potential explanation for these findings.

Finally, it is worth noting that the design of the experiments reported here naturally imposes constraints on the generality of the findings. Each experiment used fairly simple visual and auditory stimuli, assessed a very limited number of fluency and preference measures, and involved a group of young English-speaking participants. Future work should extend the paradigm to include more complex and/or real-world-like objects and sounds. Fluency could be assessed through measures other than RT, such as judgements of learning (Rhodes & Castel, 2008), and objects could be rated not just for liking but also for other relevant features such as prettiness (Reber et al., 1998) or interest (Flavell et al., 2020). Indeed, Flavell et al. (2020) show that, although increased perceptual fluency led to higher ratings when objects were assessed for liking, harder-to-perceive objects were rated more highly for interest. This suggests that the disfluent yet rewarding processing associated with successful perception of more challenging stimuli can override perceptual fluency effects for some judgements, indicating a complex and multi-faceted relationship between fluency and preference measures that may or may not replicate crossmodally. A broader range of participants, including older adults and those from non-WEIRD (Western, Educated, Industrialised, Rich and Democratic) populations (Henrich et al., 2010), should also be included. What is clear for now, however, is that the perceptual fluency of a stimulus in one domain has the potential to affect preference for a stimulus in another domain. In other words, it is possible to influence a perceiver’s judgement of an object without altering any of the physical characteristics of the object itself. While the limits of this effect and its underlying mechanisms remain to be established, such an effect has potentially important implications for a range of real-world scenarios which target preference and attitude change, such as design, advertising and healthcare. For example, McKean et al. (2020) showed that perceptual fluency manipulations within a visual task – in combination with inhibition techniques – could affect later preferences for real-world food items. If such effects could be obtained crossmodally, or indeed strengthened through simultaneous cues in a variety of perceptual domains, powerful new tools could be created to improve health and wellbeing by influencing individual preferences.

## Data Availability

Data, stimuli and analysis code are available via the Open Science Framework at https://osf.io/hsgyr/.
